# CD2 Is a Novel Immune-Related Prognostic Biomarker of Invasive Breast Carcinoma That Modulates the Tumor Microenvironment

**DOI:** 10.3389/fimmu.2021.664845

**Published:** 2021-04-23

**Authors:** Yanzhu Chen, Zhishang Meng, Lin Zhang, Feng Liu

**Affiliations:** ^1^ Department of Radiation Oncology, Hunan Cancer Hospital and The Affiliated Cancer Hospital of Xiangya School of Medicine, Central South University, Changsha, China; ^2^ Department of Ophthalmology, The Second Xiangya Hospital, Central South University, Changsha, China

**Keywords:** CD2, breast invasive carcinoma, tumor microenvironment, ImmuneScore, tumor immunology

## Abstract

Female breast cancer (BCa) is the most commonly occurring cancer worldwide. The tumor microenvironment (TME) plays an essential role in tumor invasion, angiogenesis, unlimited proliferation, and even immune escape, but we know little about the TME of BCa. In this study, we aimed to find a TME-related biomarker for BCa, especially for invasive breast carcinoma (BRCA), that could predict prognosis and immunotherapy efficacy. Based on RNA-seq transcriptome data and the clinical characteristics of 1222 samples (113 normal and 1109 tumor samples) from The Cancer Genome Atlas (TCGA) database, we used the ESTIMATE algorithm to calculate the ImmuneScore and StromalScore and then identified differentially expressed genes (DEGs) between the high and low ImmuneScore groups and the high and low StromalScore groups. Thereafter, a protein–protein interaction (PPI) network analysis and univariate Cox regression analyses of overall survival were used to identify potential key genes. Five candidate genes were identified, comprising CD2, CCL19, CD52, CD3E, and ITK. Thereafter, we focused on CD2, analyzing CD2 expression and its association with survival. CD2 expression was associated with tumor size (T stage) to some extent, but not with overall TNM stage, lymph node status (N stage), or distant metastasis (M stage). High CD2 expression was associated with longer survival. METABRIC data were used to validate the survival result (n = 276). Gene set enrichment analysis (GSEA) showed that the Kyoto Encyclopedia of Genes and Genomes (KEGG) pathways that were significantly associated with high CD2 expression were mainly immune-related pathways. Furthermore, CD2 expression was correlated with 16 types of tumor-infiltrating immune cells (TICs). Hence, CD2 might be a novel biomarker in terms of molecular typing, and it may serve as a complementary approach to TNM staging to improve clinical outcome prediction for BCa patients.

## Introduction

According to the latest global cancer data published by the International Agency for Research on Cancer (IARC), female breast cancer (BCa) is the most commonly occurring cancer worldwide (1). BCa patients’ treatment should be decided by a multidisciplinary team, taking Karnofsky Performance Status (KPS), molecular subtype, and primary tumor burden into account (2). Although studies have shown that systemic plus local therapies are critically involved in the management of patients with BCa (3), many clinicopathological factors influence the response rates among these patients. Although the American Joint Committee on Cancer/Union for International Cancer Control (AJCC/UICC) classification can be used to estimate the tumor burden and prognosis of BCa patients, clinical outcomes do not always relate to the tumor histological stage (4–6). Research has indicated that using the TME-related ImmuneScore and the proportions of tumor-infiltrating immune cells (TICs) in the tumor microenvironment (TME) may improve the prediction of several cancer prognosis (7, 8).

It is widely accepted that the TME plays an important role in tumor invasion, angiogenesis, unlimited proliferation, and even immune escape (9–11). The BCa TME is composed of heterogeneous immune and stromal cells, which play vital roles in regulating both BCa initiation and progression and the clinical responses to therapies (12). The IMpassion130 trial showed that atezolizumab plus nanoparticle albumin-bound paclitaxel improves overall survival (OS) in triple-negative PD-L1 immune cell-positive BCa (13). However, as in other solid tumors, a major limitation is the low response rate among patients to immune checkpoint inhibitor treatment. Recently, in colorectal cancer, the TME-related ImmuneScore has been shown to be an effective indicator of cancer recurrence, metastasis, and prognosis (7). Moreover, an international consensus has been reached regarding the effectiveness of using the ImmuneScore to classify colorectal cancer (6). Unfortunately, little is known about the BCa TME, but there is much interest in identifying novel prognostic and therapeutic biomarkers across the distinct pathomolecular subtypes of BCa.

Here, we aimed to find an immune-related biomarker for BCa that can predict prognosis and immunotherapy efficacy. In the era of big data, bioinformatic analysis provides a powerful tool for data mining. Using The Cancer Genome Atlas (TCGA) database, along with several bioinformatic algorithms and tools, we explored TME-related genes. Further validation and analysis confirmed that CD2 might be useful as a novel biomarker in BCa.

## Materials and Methods

### Data Preparation and Estimation of ImmuneScores, StromalScores, and ESTIMATEScores

The transcriptome profiles (HTSeq Fragments Per Kilobase of transcript per Million mapped reads [FPKM]) and corresponding clinical information on invasive breast carcinoma (BRCA) patients were downloaded from the Genomic Data Commons Data Portal of TCGA (https://cancergenome.nih.gov/) on January 10, 2021. The transcriptome data involved 1109 tumor and 113 normal tissues. The “ESTIMATE” R package was used to assess immune infiltration (based on the ImmuneScore, StromalScore, and ESTIMATEScore) using the transcriptome data. As a result, 1099 tumor samples with immune infiltration scores and clinical data were obtained. After excluding cases without follow-up data or with follow-up time <1 day, 1089 samples with both immune infiltration scores and clinical data were obtained.

### Associations of ImmuneScores, StromalScores, and ESTIMATEScores With Survival and Clinicopathological Characteristics

The samples were divided into two groups according to the medians of the ImmuneScores, StromalScores, and ESTIMATEScores. OS was used as the primary prognostic endpoint, and Kaplan–Meier survival curves were created using the “survival” and “survminer” R packages. The log-rank test was used to compare the subgroups, with *p* < 0.05 being considered significant. Additionally, the Kruskal–Wallis rank sum test was used to assess the associations between the scores and TMN staging using the “ggpubr” R package.

### Identification of TME-Related DEGs

DEGs were identified between the high and low ImmuneScore groups and between the high and low StromalScore groups (14). The “limma” R package was used to perform the differential expression analysis (15). The criteria for DEG identification were false discovery rate (FDR)-adjusted *p* < 0.05 and |log2(fold change)|>1. The results were plotted in volcano plots using the “ggplot2” R package.

### Gene Ontology and Kyoto Encyclopedia of Genes and Genomes Enrichment Analyses

Gene ontology (GO) functional enrichment and Kyoto Encyclopedia of Genes and Genomes (KEGG) pathway enrichment analyses of the DEGs were performed using the “clusterProfiler”, “enrichplot”, and “ggplot2” R packages (*p* < 0.05, *q* < 0.05) (16, 17). The results were visualized using bubble and circos diagrams.

### Protein–Protein Interaction (PPI) Network Construction

The STRING database (http://string-db.org) was used to assess the DEG-encoded proteins and PPI information. DEGs with a confidence score > 0.9 were chosen to build the network. The results were visualized using Cytoscape v3.8.2 (18). The cytoHubba plugin in Cytoscape was used to perform modular analysis, and the top 30 significant genes were selected according to the multi-network clustering (MNC) method.

### Univariate Cox Regression Analyses

To identify which of the DEGs were associated with OS, we performed univariate Cox regression analyses using the “survival” R package. The DEGs with *p* < 0.05 were displayed using forest plots.

### CD2 Differential Expression and Survival Analyses

The Wilcoxon rank sum test was used to compare CD2 mRNA expression between BRCA tissues and normal using the “beeswarm” and “ggpubr” R packages. Survival analyses of low and high CD2 expression were performed using the “survival” R package. Differential CD2 protein expression between BRCA and normal tissues was determined using immunohistochemistry (IHC) results from the Human Protein Atlas (HPA) database (https://www.proteinatlas.org/). Spearman’s correlation analysis was used to determine the correlation between CD2 expression and tumor mutation burden (TMB) using the “ggstatsplot” R package. External validation of the survival analysis was performed using the METABRIC data cohort using Breast Cancer Gene-Expression Miner v4.5 software (bc-GenExMiner v4.5; http://bcgenex.centregauducheau.fr) (19). The Kruskal–Wallis rank sum test was used to assess the associations between CD2 expression and TMN staging using the “ggpubr” R package.

### Gene Set Enrichment Analysis

Gene Set Enrichment Analysis (GSEA) was performed using GSEA v4.1.0 software to identify the biological pathways associated with high and low CD2 expression. Pathways with nominal *p* < 0.05 were considered significantly enriched.

### Evaluation of Immune Cell Infiltration

The proportions of 22 immune cell types (i.e., TICs) in each of the 1099 tumor samples (with immune infiltration scores) were calculated using the CIBERSORT algorithm (20) and visualized using bar charts. The proportions were then compared between tumor tissues with low or high CD2 expression using Wilcoxon rank sum test, and Pearson’s correlation was assessed between the proportions and CD2 expression. The results were presented in violin and scatter plots, respectively, using the “vioplot”, “ggplot22”, and “ggpubr” R packages.

## Results

### Analysis Process of Patients and Data Sets

**Figure 1:**
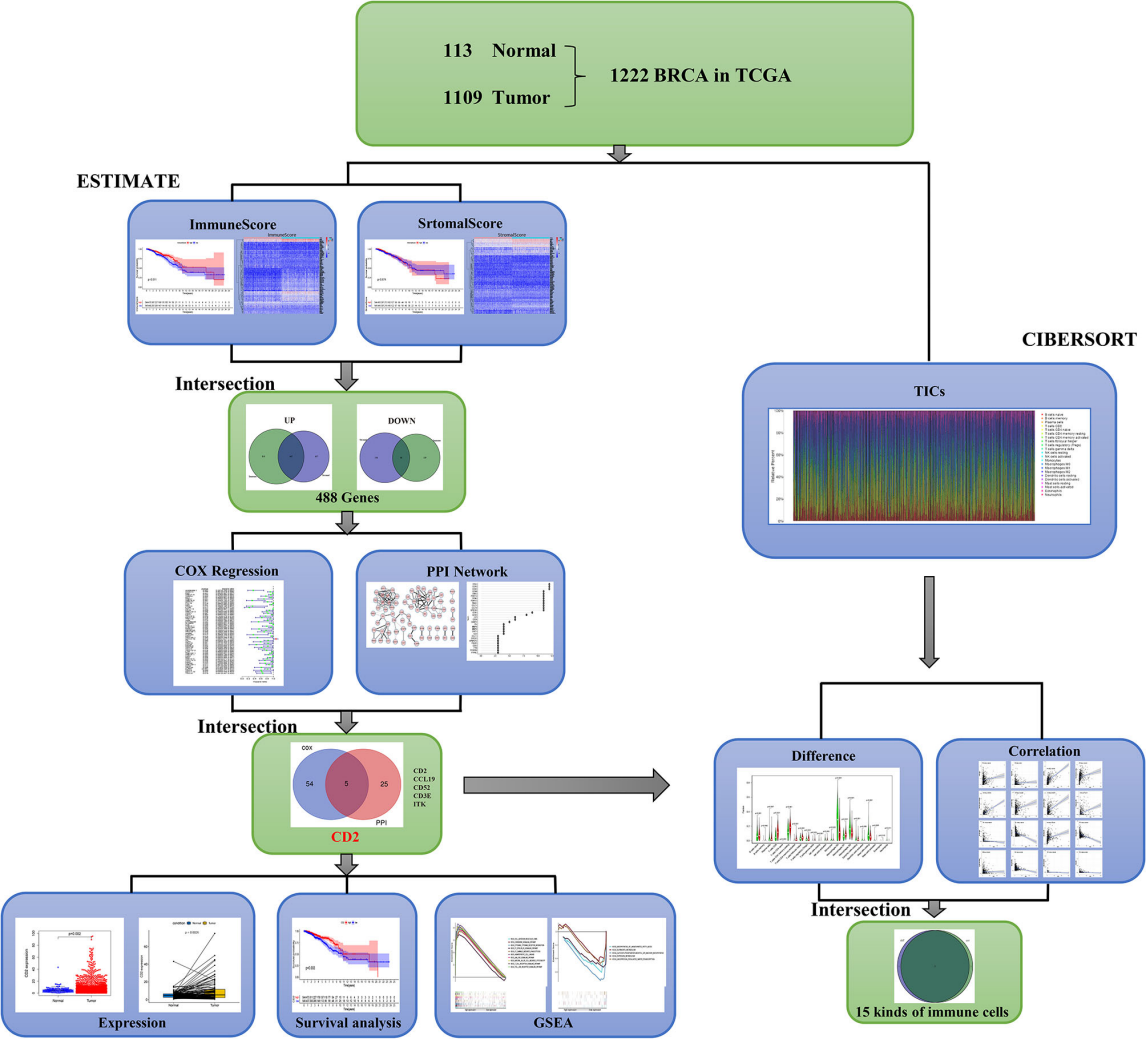
Analytical flowchart of this study.

A brief flowchart of our study is shown in **Figure 1**. Based on the transcriptome data and clinical characteristics related to 1222 samples (113 normal and 1,109 tumor samples) from the TCGA database, ImmuneScore and StromalScore were calculated by ESTIMATE algorithms to assess the immune and stromal components, and CIBERSORT algorithms were used to estimate the proportions of TICs. DEGs were identified between the low and high ImmuneScore and StromalScore groups. PPI network analysis and univariate Cox regression analyses of the DEGs were performed. Next, CD2, CCL19, CD52, CD3E, and ITK were identified based on the intersection of the hub genes in the PPI network (based on the cytoHubba plugin in Cytoscape) and the 59 significant genes in the univariate Cox regression analyses. We then analyzed CD2 further, including analyses of expression, survival, and clinicopathological characteristics, GSEA, and correlation with the proportions of TICs.

### ImmuneScore Is Associated With BRCA Patients’ Prognosis

**Figure 2:**
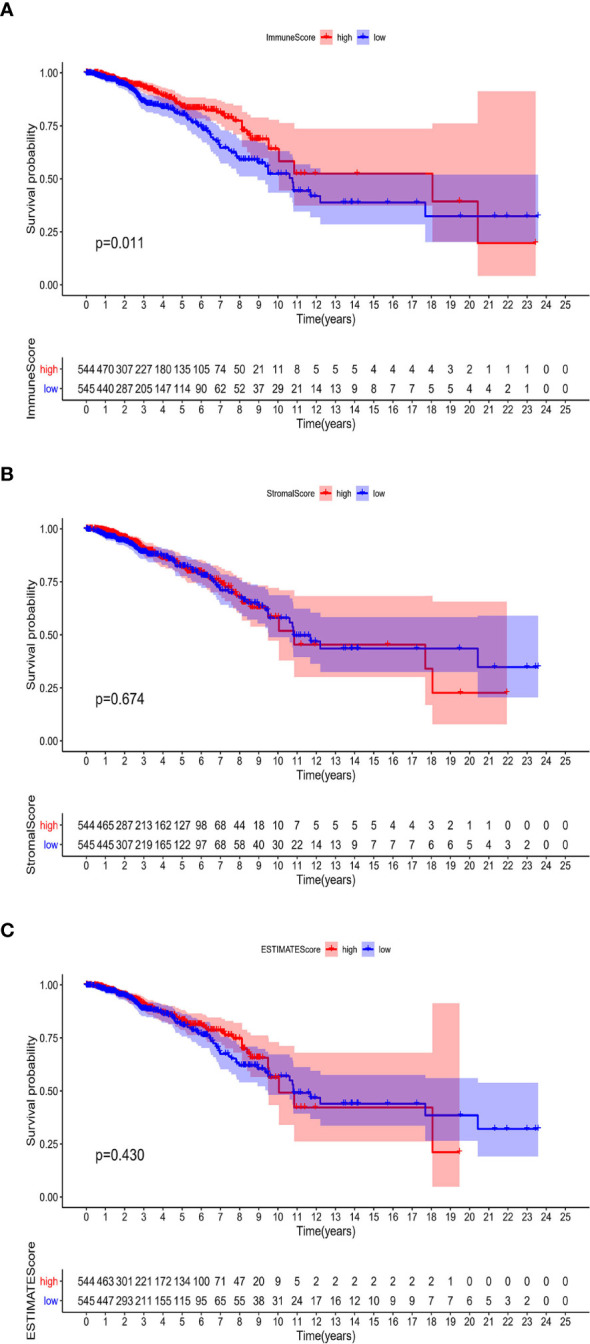
Associations of ImmuneScore, StromalScore, and ESTIMATEScore with survival among BRCA patients. Kaplan–Meier survival analyses of BRCA patients with low and high **(A)** ImmuneScores (*p* = 0.011), **(B)** StromalScores (*p* = 0.674), and **(C)** ESTIMATEScores (*p* = 0.430).

The associations of the immune infiltration scores with BRCA patients’ prognosis were assessed. For each of the scores (ImmuneScore, StromalScore, and ESTIMATEScore), half of the 1089 tumor samples (with complete clinical data) were classified into the high score group (544 cases) and the other half were classified into the low score group (545 cases). Thereafter, Kaplan–Meier survival curves were constructed. Patients with a high ImmuneScore had better OS than those with a low ImmuneScore (log-rank test *p* = 0.011), and a longer mean OS time (**Figure 2A**). However, StromalScore and ESTIMATEScore were not significantly associated with OS (**Figures 2B, C**). These results reveal that the immune components in the TME are potentially positive factors in the prognosis of BRCA patients.

### Associations of ImmuneScore, StromalScore, and ESTIMATEScore With TNM Staging Among BRCA Patients

**Figure 3:**
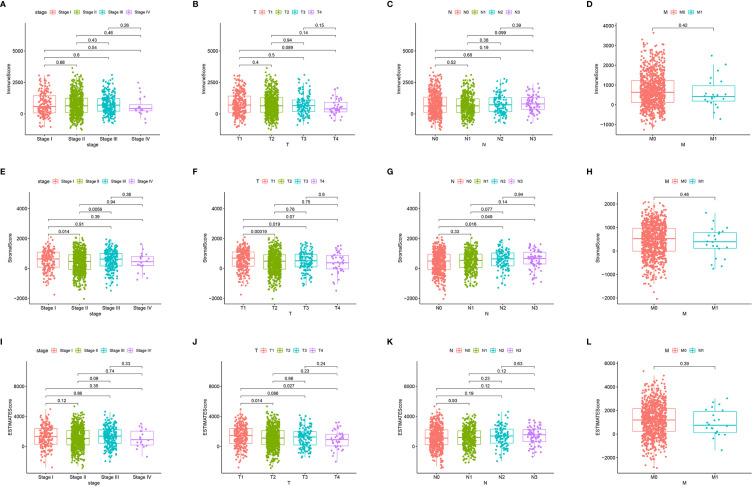
Associations of ImmuneScore, StromalScore, and ESTIMATEScore with TNM staging. Kruskal–Wallis rank sum tests of the associations of **(A–D)** TME immune components, **(E–H)** TME stromal components, and **(I–L)** TME immune and stromal components with TNM staging.

First, we assessed the number of immune and stromal components separately, and then analyzed the associations of ImmuneScore, StromalScore, and ESTIMATEScore with primary tumor size (T stage), regional lymph node status (N stage), distant metastasis (M stage), and overall stage of BRCA patients from the TCGA database (**Figure 3**). ImmuneScore was not significantly associated with T stage, N stage, M stage, or overall stage (**Figures 3A–D**). However, the StromalScore analysis showed that the prevalence of stromal components was higher in Stage I vs II (*p* = 0.014) and Stage III vs II (*p* = 0.0056) (**Figure 3E**), and StromalScore was also significantly associated with T stage and N stage (**Figures 3F, G**) but not with M stage (**Figure 3H**). Additionally, ESTIMATEScore was significantly associated with T stage (**Figure 3J**) but not with N stage, M stage, or overall stage (**Figures 3I, K, L**). These results indicate that the stromal components of BRCA samples are probably clinically associated with its biological behavior, such as infiltration and metastasis.

### DEGs Between the High and Low ImmuneScore and StromalScore Groups Were Mainly Immune-Related Genes

**Figure 4:**
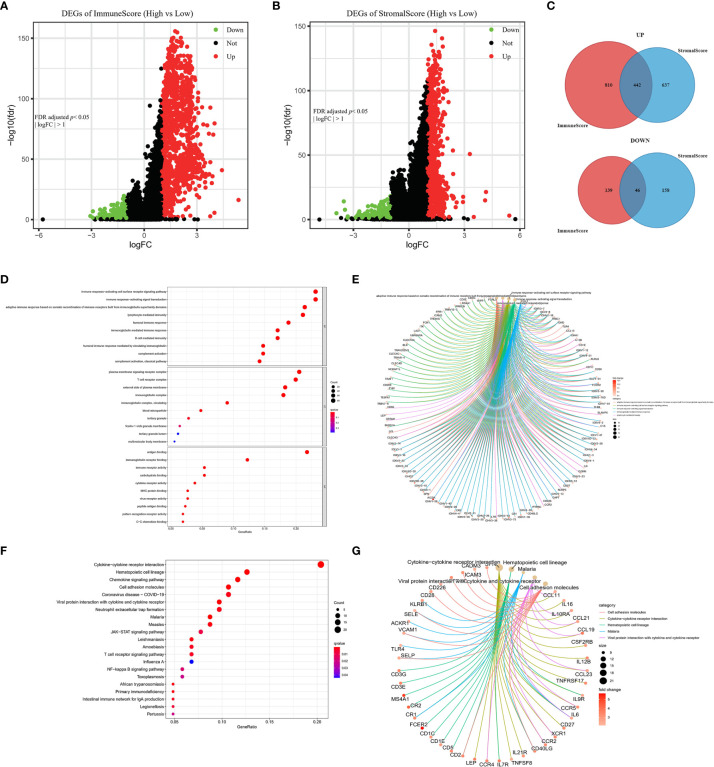
Volcano plots, Venn diagrams, and GO and KEGG enrichment analyses of differentially expressed genes (DEGs). Volcano plots of significant DEGs (FDR-adjusted *p* < 0.05, |log FC| > 1) between high and low **(A)** ImmuneScore and **(B)** StromalScore groups. **(C)** Venn diagrams of upregulated and downregulated DEGs that were shared by the ImmuneScore and StromalScore analyses. **(D, E)** GO and **(F, G)** KEGG enrichment analyses (*p* < 0.05 and *q* < 0.05).

To determine the associations of gene expression with ImmuneScore and StromalScore, we analyzed transcriptome data for 1099 BRCA patients from the TCGA database by comparing gene expression between the high and low ImmuneScore and StromalScore groups, as shown in volcano plots in **Figures 4A, B**. There were 1437 DEGs (1252 upregulated and 185 downregulated) between the high and low ImmuneScore groups, and 1283 DEGs (1079 upregulated and 204 downregulated) between the high and low StromalScore groups. Moreover, there were 488 shared DEGs (442 upregulated and 46 downregulated) among both analyses (**Figure 4C**), which may be the key factors regarding the status of the TME.

Furthermore, GO and KEGG analyses predicted the functions of these shared DEGs. The GO analysis indicated that the DEGs almost clustered in the immune-related GO terms, such as immune response-activating cell surface receptor signaling pathway, plasma membrane signaling receptor complex, and antigen binding (**Figures 4D, E**). The KEGG analysis showed that the DEGs were mainly enriched in cytokine–cytokine receptor interaction, chemokine signaling pathway, and cell adhesion molecules (**Figures 4F, G**). Thus, the DEGs seem to be related to immune activity, which illustrates that immune factors play a crucial role in the TME of BRCA patients.

### PPI Network and Univariate Cox Regression Analyses

**Figure 5:**
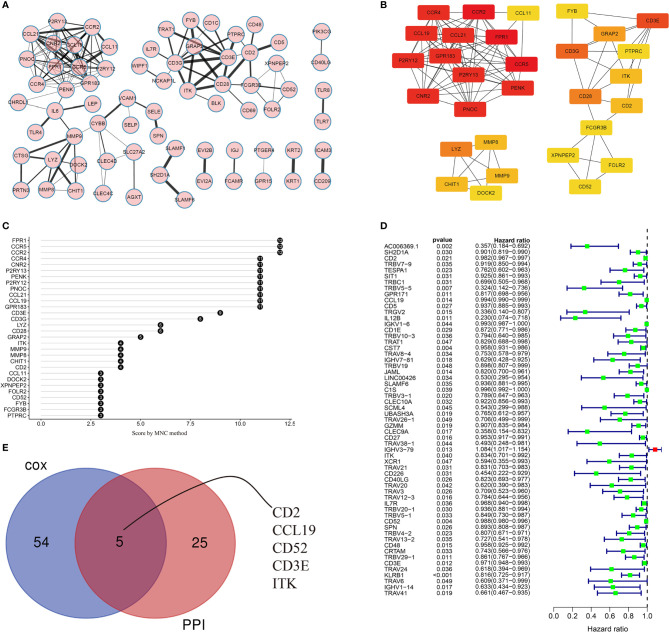
Visualization of the PPI network and univariate Cox regression analyses. **(A)** PPI network based on STRING confidence score >0.9. Thicker edges between nodes indicate stronger combined scores. **(B)** Identification of hub genes in the PPI network using the MNC algorithm. Red nodes represent genes with high MNC scores, while yellow nodes represent genes with lower MNC scores. **(C)** Lollipop diagrams of MNC scores of the top 30 genes. **(D)** Forest plot of univariate Cox regression analyses, showing genes with *p* < 0.05. **(E)** Venn diagram of the intersection between the top 30 hub genes in the PPI network and the 59 significant genes in the Cox regression analyses.

The PPI network (with 71 nodes and 145 edges) was constructed using the 488 shared DEGs based on STRING PPI confidence scores >0.9 in the STRING platform (https://string-db.org/). The results were visualized in Cytoscape v3.8.2 using EnrichmentMap (**Figure 5A**). The top 30 hub genes were then identified by the MNC algorithm using the cytoHubba plugin in Cytoscape (**Figures 5B, C**). Thereafter, we subjected the 488 DEGs to univariate Cox proportional hazard regression analyses and 59 genes were significant (*p* < 0.05) (**Figure 5D**). Five hub genes (CD2, CCL19, CD52, CD3E, ITK) were identified based on the intersection of the 30 hub genes in the PPI network and the 59 significant genes in the Cox regression analyses, as visualized in the Venn diagram (**Figure 5E**).

### CD2 Expression, Survival, and Clinicopathological Characteristic Analyses

**Figure 6:**
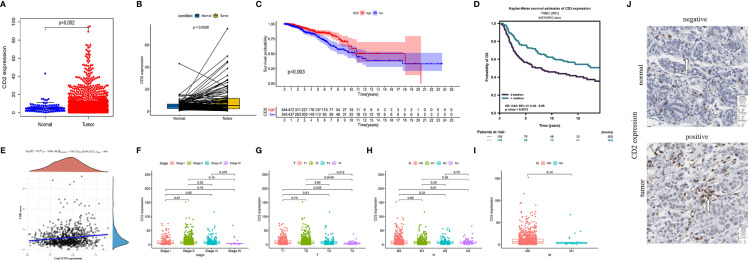
CD2 expression, survival, and clinicopathological characteristic analyses. CD2 was differentially expressed between tumor tissues and **(A)** normal tissues (Wilcoxon rank sum test *p* = 0.002) and **(B)** paired normal tissues deriving from the same one tumor patient (Wilcoxon rank sum test *p* = 0.0026). **(C)** Survival analysis of BRCA patients with low and high CD2 expression (based on median expression) in the TCGA database (log-rank test *p* = 0.003). **(D)** Survival analysis of triple-negative BCa patients with low and high CD2 expression (based on median expression) in the METABRIC database (log-rank test *p* = 0.0073). **(E)** Correlation analysis of CD2 expression and TMB (*p* < 0.001, *ρ*_Spearman_ = 0.14). **(F–I)** Kruskal–Wallis rank sum tests of the associations of CD2 expression with TNM staging. **(J)** Immunohistochemistry analysis of CD2 protein expression in normal and BRCA tissues from the Human Protein Atlas (HPA) database. Normal breast tissue – patient id: 2733, antibody: HPA003883, staining: not detected, intensity: negative, quantity: none; tumor tissue – patient id: 2015, antibody: HPA003883, staining: low, intensity: weak, quantity: >75%.

Interestingly, CD2 expression was significantly reduced in both normal and paired tissues (derived from the same patient) compared to BRCA tissues (*p* = 0.002 and *p* = 0.0026, respectively, **Figures 6A, B**). BRCA patients with low CD2 expression (based on the median CD2 expression) had significantly shorter OS time than those with high CD2 expression (**Figure 6C**). Thereafter, the survival analysis was validated using the METABRIC database (n = 276). The triple-negative BCa patients with high CD2 expression had a significantly better prognosis than patients with low CD2 expression (**Figure 6D**). CD2 expression was significantly positively correlated with TMB (*p* < 0.001, *ρ*_Spearman_ **= 0.14) (**Figure 6E**), Regarding the analysis of clinicopathological characteristics, CD2 expression was associated with tumor size to some extent (**Figure 6G**), but not with overall TNM stage (**Figure 6F**), lymph node status (**Figure 6H**), or distant metastasis (**Figure 6I**). Moreover, CD2 protein expression was higher in tumor tissues compared to normal tissues based on the HPA database (**Figure 6J**).

### GSEA of CD2

**Figure 7:**
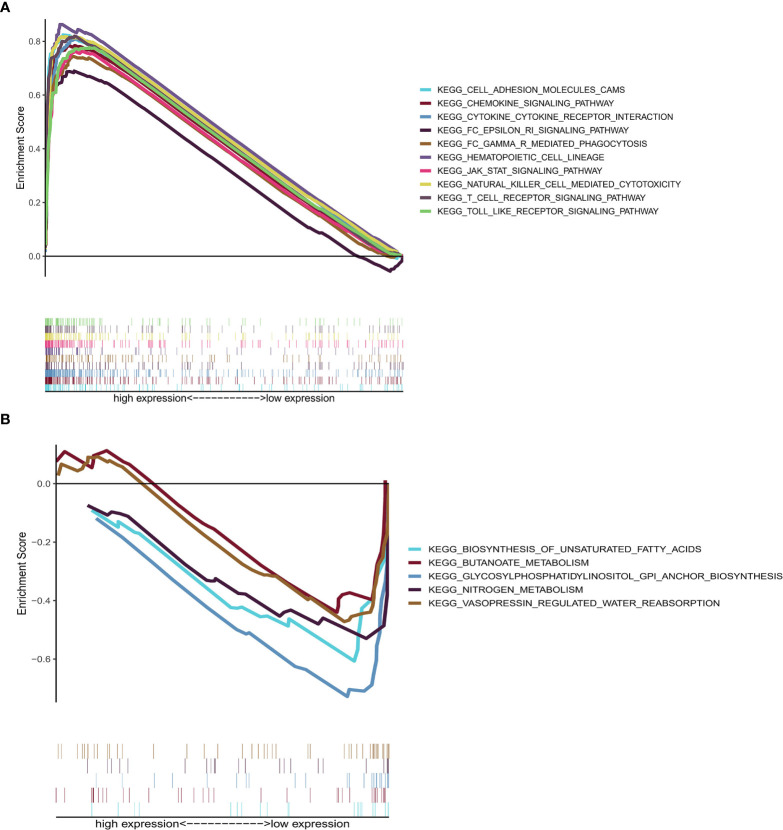
GSEA of samples with high or low CD2 expression. **(A)** Top ten significant pathways associated with high CD2 expression (nominal *p* < 0.05 and FDR-adjusted *q* < 0.05). **(B)** Top five significant pathways associated with low CD2 expression (nominal *p* < 0.05).

GSEA, using GSEA v4.1.0 software, was employed to assess the KEGG pathways that were significantly associated with high and low CD2 expression. The significant pathways associated with high CD2 expression were mainly immune-related pathways, such as cell adhesion molecules, chemokine signaling pathway, cytokine–cytokine receptor interaction, FcϵRI signaling pathway, and FcδR-mediated phagocytosis (the top ten pathways are shown in **Figure 7A**). The top five significant pathways associated with low CD2 expression were metabolic pathways, comprising biosynthesis of unsaturated fatty acids, butanoate metabolism, glycosylphosphatidylinositol (GPI)-anchor biosynthesis, nitrogen metabolism, and vasopressin-regulated water reabsorption (*p* < 0.05) (**Figure 7B**). This indicates that CD2 plays an essential biological role in the immune status of the TME.

### Correlation of CD2 Expression With TICs

**Figure 8:**
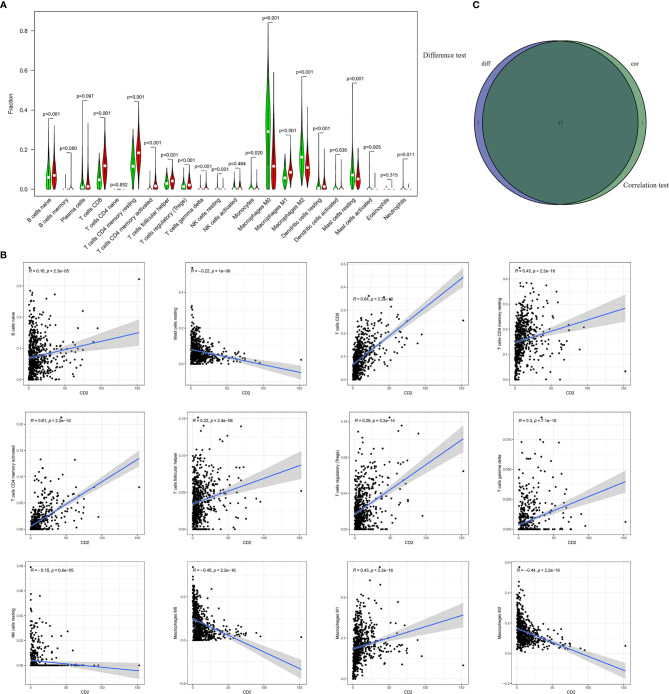
Differences in proportions of TICs between high and low CD2 expression groups and correlations of TICs with CD2 expression. **(A)** Violin plots of the proportions of 22 immune cell types in tumor tissues with low (green) or high (red) CD2 expression, compared using the Wilcoxon rank sum test. **(B)** Scatter plots showing Pearson’s correlation between the proportions of the 12 most significant TICs and CD2 expression. Blue lines denote the best-fit linear models. **(C)** Venn diagram of intersection between analyses in **(A, B)** showing that 15 TICs were shared between the analyses.

To further investigate the interplay between CD2 expression and the TME, we focused on TICs of BRCA samples using the CIBERSORT algorithm. First, the comparison of the proportions of the 22 immune cell types between the high and low CD2 expression groups revealed 16 TICs with significant differences (**Figure 8A**). Furthermore, 16 of the 22 TICs were correlated (ten positively and six negatively) with CD2 expression in BRCA cases (**Figure 8B**). Surprisingly, the intersection of the analyses showed that 15 TICs were shared between the two analyses (**Figure 8C**), which provides strong evidence for the vital role of CD2 in the TME of BRCA samples.

## Discussion

Here, we sought to identify immune-related prognostic genes involved in the TME that also influenced OS in BRCA patients. CD2 was confirmed to be associated with the immune response, so it may be a prognostic and therapeutic biomarker in BRCA.

The ESTIMATE algorithm is an integrated approach (based on gene expression) to estimate tumor purity, thereby helping to identify candidate TME-related biomarkers. Based on TCGA transcriptome data, we used the ESTIMATE algorithm to identify ImmuneScores and StromalScores. We then observed that a high ImmuneScore led to better OS, suggesting that a TME-related factor was potentially able to estimate prognosis, which coincides with previous reports (21, 22). We then identified the TME-related DEGs between the high and low ImmuneScore and StromalScore groups. GO and KEGG analyses suggest that many of these DEGs were involved in immune-related processes. To further investigate immune-related genes associated with prognosis, we constructed a PPI network using Cytoscape, and then the top 30 hub genes were identified, Thereafter, 59 of the DEGs were found to be significantly associated with OS based on univariate Cox regression analyses. We identified five key genes, comprising CD2, CCL19, CD52, CD3E, and ITK, based on the intersection between the hub DEGs in the PPI network and the OS-associated DEGs. Further analysis demonstrated that CCL19, CD3E, and ITK were not strongly associated with OS in our sample. However, previous research demonstrated that CD52 can play a significant prognostic role in BRCA patients (23). CD2 is an essential component in the immunological synapse, the actin cytoskeleton, and agonistic cell signaling, and it is expressed on the surface of T, NK, and dendritic cells (24). Therefore, we selected CD2 for further investigation.

We found that compared to normal and paired tissues, CD2 was highly expressed in BRCA samples, which is consistent with other studies (25, 26). Our data also indicated that high CD2 expression was associated with better prognosis and high TMB in BRCA patients. For the first time, we report that CD2 is involved in the TMB of BRCA, providing new insights into immune therapy for BCa. In multiple clinical studies of solid tumors, high TMB indicates better immunotherapy efficacy (27–29). A recent study showed that CD2 downregulation may attenuate antitumor T cell responses in colorectal, endometrial, and ovarian cancer, and even offset the benefit of PD-1 immunotherapy (24). In addition, some studies have reported that targeting CD2 using a monoclonal antibody can maintain immunosuppression in organ transplantation patients and reduce organ rejection (30, 31). And organ transplant patients taking immunosuppressants may have an increased risk of cancer (32). Further investigations about the mechanisms of CD2-induced immune activities will be important for a full understanding of immune-tolerance and immunotherapy strategy.

In an additional analysis of the correlation between CD2 expression and TNM staging, we found that CD2 was involved in tumor invasion but had no association with lymph node or distant metastasis. In contrast, a study of human epidermal growth factor receptor 2 (HER2) + BCa found that CD2 expression was associated with distant metastasis-free survival (25). The different conclusions may be associated with the different inclusion and exclusion criteria. More research is needed to confirm these findings. Interestingly, in the external validation analysis, we found that high CD2 expression led to a significantly better prognosis in triple-negative BCa patients. Furthermore, a previous study indicated that in hormone receptor-negative (HR-)/HER2+ BCa, high CD2 expression led to a significantly longer OS (25), and our findings provide novel evidence for the same association in another BCa subtype.

Furthermore, we performed GSEA and found that high CD2 expression was associated with immune-related signaling pathways, which means that the CD2 expression impacts the TME in BRCA patients. Studies have demonstrated that CD2 immunoregulation involving T and NK cell activation (33). We also identified the proportions of 22 immune cell types in BRCA samples using the CIBERSORT algorithm. There were 16 shared immune cell types between (1) the analysis of differences in the proportions of TICs between high and low CD2 expression groups and (2) the correlations of TICs with CD2 expression in BRCA. Among these 16 cell types, CD2 expression was positively associated with ten (naive B cells, plasma cells, CD8 T cells, resting memory CD4 cells, activated memory CD4 cells, follicular helper T cells, regulatory T cells, gamma delta T cells, M1 macrophages, and resting dendritic cells) and negatively associated with six (resting NK cells, M0 macrophages, M2 macrophages, resting mast cells, activated mast cells, and neutrophils). Our findings confirmed that CD2 plays important roles in the TME of BRCA. Previous studies demonstrated that dense infiltration of certain immunocytes (CD8T cells, resting memory CD4 cells, activated memory CD4 cells, and M1 macrophages) indicates a favorable prognosis (34–36). Conversely, a low density of resting NK cells, M0 macrophages, and M2 macrophages indicate a poor clinical outcome among patients with hematological malignancies and lymphoma (37, 38). Our study supports these previous conclusions. Recent research has demonstrated that the immune system influences tumor development, with NK cells with more inherited gene defects being more likely to lead to tumor development and these inherited gene defects being associated with TME subtypes (39). The relationship between CD2 and NK cells requires further study.

Our findings theoretically indicate that increasing the density of CD2 in BRCA tissue might improve immunotherapy efficacy by increasing the tumor immunogenicity and the antitumor immune responses. In particular, the immunomodulatory effects of CD2 may play a crucial role in delaying disease progression. Thus, CD2 may be useful as an immunomodulatory agent for BCa management.

## Conclusion

Our study indicates that CD2 exhibits immunity-related activity in the TME of BCa, so it might influence the biological behavior and phenotype of BCa. This helps to explain the diverse clinical outcomes among tumors of the same TNM stage treated with the same strategy. As a result, CD2 might be a novel indicator in terms of molecular typing, and it may serve as a complementary approach to TNM staging to improve clinical outcome prediction. Improving the identification of immunotherapy-sensitive BCa patients will help us to move precision medicine forward.

## Data Availability Statement

The datasets generated and analyzed during this study are available in the TCGA database (https://portal.gdc.cancer.gov).

## Author Contributions

YC, ZM, and LZ conceptualized the study. YC and ZM wrote the manuscript and constructed the figures. YC, ZM, and LZ performed the data analysis. FL reviewed and edited the manuscript. LZ and FL acquired funding for the project. All authors contributed to the article and approved the submitted version.

## Funding

This study was funded by Hunan Provincial Science and Technology Department (Hunan Provincial Natural Science Foundation of China; grant number 2016JJ6088), Beijing Hope Run Special Fund of Cancer Foundation of China (grant number LC2016W05), Health Commission of Hunan Province (grant number B2016048), and Science and Technology Project of Changsha City (grant number kq1701042).

## Conflict of Interest

The authors declare that the research was conducted in the absence of any commercial or financial relationships that could be construed as a potential conflict of interest.
